# 456. Within-host Viral Evolution and Neutralizing Antibody Response in Immunocompromised Patients with Persistent *SARS-CoV-2* Infection Receiving B Cell Depleting Treatment

**DOI:** 10.1093/ofid/ofad500.526

**Published:** 2023-11-27

**Authors:** Sangmin Ahn, Yongseop Lee, Min Han, Jung Ah Lee, Jung Ho Kim, Su Jin Jeong, Nam Su Ku, Jun Yong Choi, Joon-sup Yeom, Jin Young Ahn

**Affiliations:** Yonsei University College of Medicine, seoul, Seoul-t'ukpyolsi, Republic of Korea; Yonsei University School of Medicine, Seoul, Seoul-t'ukpyolsi, Republic of Korea; Yonsei University School of Medicine, Seoul, Seoul-t'ukpyolsi, Republic of Korea; Yonsei University College of Medicine, seoul, Seoul-t'ukpyolsi, Republic of Korea; Yonsei University College of Medicine, seoul, Seoul-t'ukpyolsi, Republic of Korea; Yonsei University College of Medicine, seoul, Seoul-t'ukpyolsi, Republic of Korea; Division of Infectious Diseases, Department of Internal Medicine, Yonsei University College of Medicine, Seoul, Seoul-t'ukpyolsi, Republic of Korea; Yonsei University College of Medicine, seoul, Seoul-t'ukpyolsi, Republic of Korea; Division of Infectious Diseases, Department of Internal Medicine, Yonsei University College of Medicine, Seoul, Seoul-t'ukpyolsi, Republic of Korea; Yonsei University College of Medicine, seoul, Seoul-t'ukpyolsi, Republic of Korea

## Abstract

**Background:**

There are reports of immunocompromised patients who have received B cell depleting treatment becoming infected with severe acute respiratory syndrome coronavirus-2 (SARS-CoV-2) and sustaining viral shedding for months or longer. Detailed information on intrahost viral evolution in SARS-CoV-2 prolonged infection and neutralizing antibody response against SARS-CoV-2 is limited.

**Methods:**

We analyzed three patients with prolonged SARS-CoV-2 infection who had been treated with B cell depleting treatment for follicular lymphoma and diagnosed their first COVID-19 between April and June 2022 during the Omicron BA.2. sublineage outbreak in South Korea. Sequential sampling of nasopharyngeal swabs and sputum specimens was performed for SARS-CoV-2 PCR and viral culture during SARS-CoV-2 infection. Whole genome sequencing (WGS) of SARS-CoV-2 was performed twice for each patient in respiratory samples two months apart obtained during the Omicron BA.5 sublineage outbreak in Korea. Sequential blood sampling was also performed for evaluating neutralizing antibody response against wild-type, BA.2 and BA. 5 using plaque reduction neutralization tests (PRNT) during SARS-CoV-2 infection.

**Table 1.**
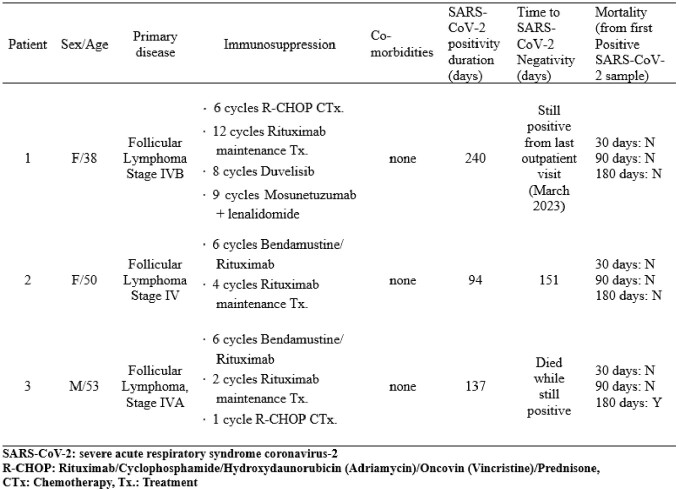
Characteristics of persistent SARS-CoV-2 infected patients.

**Figure 1.**
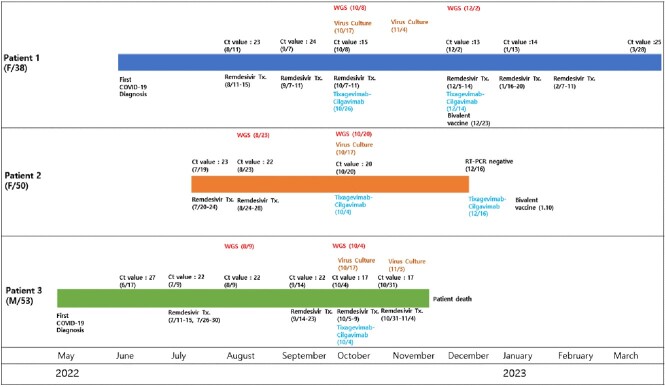
Timeline of the clinical progress and performed analysis of persistent SARS-CoV-2 infected patients.

The colored bar graphs for each patient represent the duration of viral shedding. All three patients received remdesivir, patient 1 and patient 2 received two doses of tixagevimab-cilgavimab (total 600mg/600mg), and patient 3 received one dose (total 300mg/300mg). Ct, cycle threshold; WGS, Whole Genome Sequences; Tx., treatment

**Results:**

Three patients showed sustained positive results of SARS-CoV-2 PCR for six to nine months and had viable viruses in the virus culture. All the tested samples were determined as Omicron sublineage variant BA.2.3 by WGS suggesting the persistent infection with the same strain rather than re-infection of the new strain. In addition, WGS performed on two-month interval samples showed several mutations in the S gene and ORF1ab genes, confirming within-host viral evolution. In the results of PRNT, all patients showed very low neutralizing antibody response against wild-type virus, BA.2 and BA.5 despite the prolonged course of SARS-CoV-2 infection. However, after receiving Tixagevimab-cilgavimab, there was a rapid increase in neutralizing antibody response.

**Figure 2.**
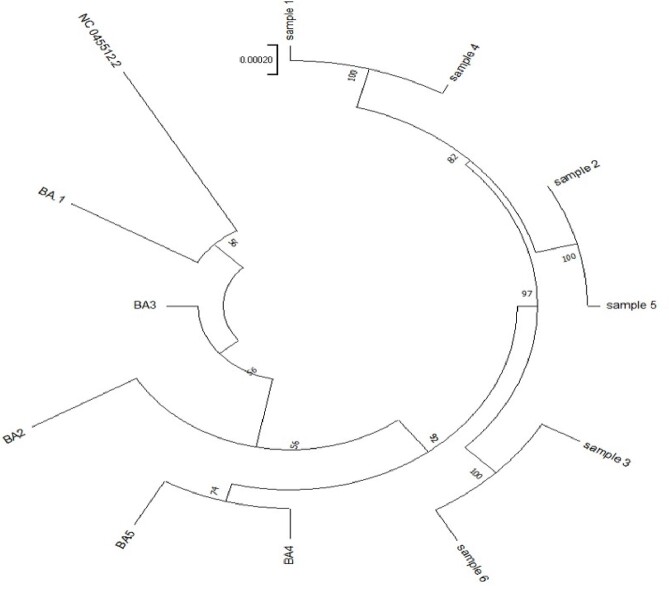
Phylogenetic analysis of viral sequences from nasopharyngeal swabs of patients with persistent SARS-CoV-2 infection

Viral sequencing was performed on each patient at two-month intervals (between August and December 2022), and a total of six samples were used to construct the phylogenic tree. Patient 1's whole genome sequencing samples are Samples 1 (early) and 2 (late), Patient 2's are Samples 3 (early) and 4 (late), and Patient 3's are Samples 5 (early) and 6 (late). All six samples were analyzed as a lineage of the dominant omicron sub-lineage variant BA.2.3 at the time of the patients' first COVID-19 diagnosis.

**Table 2.**
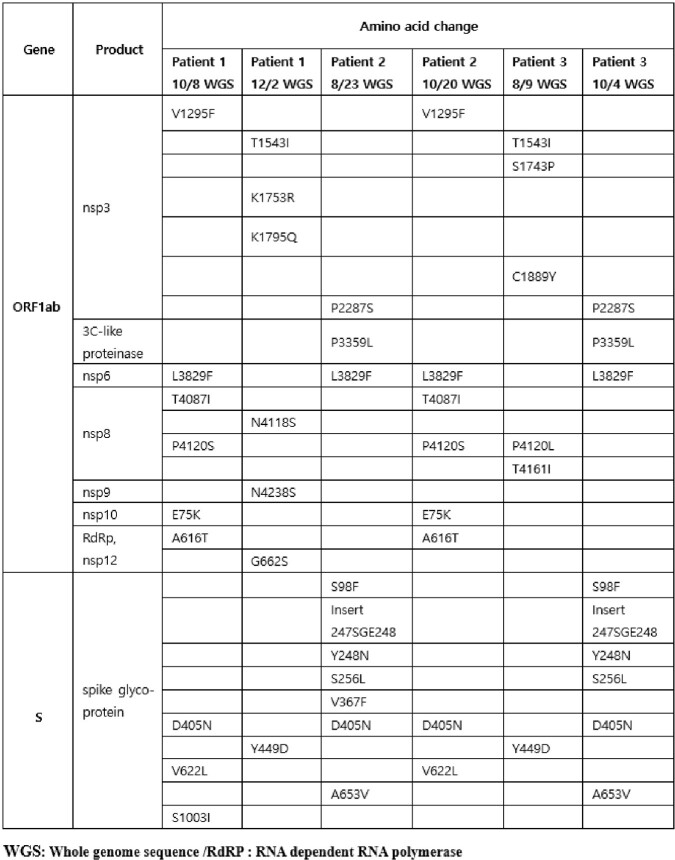
Amino acid changes in the viral whole genome sequence of patients with persistent SARS-CoV-2 infection

**Table 3.**
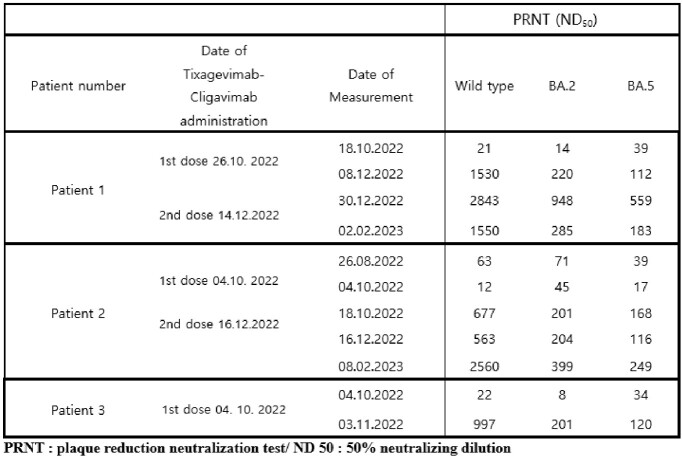
Neutralizing antibody response after Tixagevimab-Cligavimab administration in patients with persistent SARS-CoV-2 infection

**Conclusion:**

Persistent SARS-CoV-2 infection in patients receiving B-cell depleting treatment showed sustained viral shedding and low neutralizing antibody response can cause intrahost viral evolution. Increased neutralizing antibody response against SARS-CoV-2 can be achieved by receiving Tixagevimab-cilgavimab in these patients.

**Disclosures:**

**All Authors**: No reported disclosures

